# Annotations

**Published:** 1888-12-15

**Authors:** 


### ANNOTATIONS.

"Bread, or a Stone?"

WHEN a typical Briton wishes to understand a thing and to do it, there is no man in the world who has clearer eyes, better hands, and a more resolute will. But when he makes up his mind that he will not see it and will not do it, there is not in all the created world a blinder owl or a more obstinate mule. Six men went to the Mansion House on Monday, commissioned by a meeting of their fellows on Clerkenwell Green, to ask the Lord Mayor to help London men who were out of work to find employment. It was not a very out-of-the-way request. There is happily nothing that strikes us as unnatural in needy men asking powerful men to help them. It is, indeed, the very thing they should do. They might steal by preference, or set fire to a street, or blow up a church, or a chapel, or a gin-palace with gunpowder. In any of these ways they would compel attention, and what is more, would secure lodging, clothing, and food. The Lord Mayor of London is usually a practical man. In most cases he has proved his practicability by making for himself a fortune. He will tell you he is none of your "bloated aristocrats," who "neither toil nor spin." He is the architect of his own fortune. Not seldom he has known the pinch of poverty in his earlier days. What has this practical man, as represented at present by Lord Mayor Whitehead, to say to men who have practical experience of starvation, and who want some practical help? "*Non possumus*," only he does not chant it like a monk in a dead language. If there is one platitude more time-disgraced than another it is that starving men ought not to be provided with work out of the rates. Are they provided with poorhouses out of the rates? Are they provided with prisons out of the rates? Are they provided with ropes out of the rates, when desperation has driven them to capital crimes, and the hangman is called in? Do the rates pay the hangman's wages? Will the Lord Mayor sit down in his parlour at home, or in his apartment at the Mansion House, and point out exactly and precisely, so that there may be no possibility of doubt about it, where lies the difference in principle between finding work out of the rates, and finding a poorhouse? Does the Lord Mayor, or do other people who satisfy their minds and consciences with platitudes which they think are the offspring of political economy: Do they ever in their own business, when necessity demands it, venture to act on their own common sense, without reference to political economy? Mr. Gladstone relegated political economy to Saturn on a famous occasion. We would relegate it to a much more uncomfortable place than that, if we could believe it taught us to compel men to become criminals or paupers before it permitted them to be helped by work from the rates. The parish is the unit. If the parish consisted of ten persons, and one of them were to have his leg cut off so that he could no longer plough, would it not be both cheaper and better to let him chop wood, or clean boots, or make clothes for the other nine, each of them contributing to his maintenance, than to give him no work at all, but to build a poorhouse or a prison for him, and then to keep him there in idleness, and to ruin his character at the same time? If it would be better in a parish of ten to find work for the workless, it would be better also in a parish of a hundred, or of a hundred thousand, or in a country of a hundred millions. The real fact is this, and it is foolish and worse to hide it—nobody in power, either in the parish, in the city, or in the state, has the clear and comprehensive intelligence to devise a working scheme. They will not confess their want of capacity. They will rather call up a wretched ghost from the vasty deep of an exploded political economy, and blame the ghost. Lord Mayor Whitehead has now his opportunity. He did not make a good beginning on Monday. "Fine words butter no parsnips." Platitudes may produce flatulent distension, but they do not satisfy the cravings of hunger. Is there no man of intelligence and will in all this vast powerful, and splendid Empire who can solve the problem of the willing, but workless, poor?

Boycotting Sir Morell Mackenzie at Edinburgh.

THE students of Professor Thomas Fraser's class of materia medica at the University of Edinburgh, if correctly reported, have made an energetic demonstration against the "boycotting" of Sir Morell Mackenzie in that city by their professors and the medical profession. The class of materia medica at Edinburgh was for many years, as old Edinburgh men well know, the class of the Nestor of Scottish medicine, Sir Robert Christison. It is a senior class, and inherits traditions, coming down from Christison's time, of high conduct and broad tolerance. It consists of two or three hundred of the picked men of the Edinburgh University, and therefore of the very flower of the medical students' world. Its behaviour may properly be taken as an indication of the feeling of the most cultured class of medical students at the present crisis. Now, it is of some importance for the medical professors of the Edinburgh University and for the whole profession in that town, to ask themselves why there is so conspicuous a difference between them and the young men who are entering the profession on the present occasion. Apparently there has been a definite agreement on the part of the leaders of Edinburgh medicine, that Sir Morell Mackenzie should be boycotted. There are some of those leaders who know well that the "boycotting" of a professional brother is a serious step. Sir Douglas Maclagan, Professor Granger Stewart, Dr. Affleck, and others are men of first-rate intelligence and high character. They know that important moral questions lie at the root of professional ostracism. Can they justify to themselves on moral grounds the severe blow they have attempted to strike at Sir Morell Mackenzie? We do not say they cannot, but we ask if they can. Tried by any standard of philosophic morals—and all the cultured physicians of Edinburgh know well how to use such standards—tried so, we ask, has Sir Morell Mackenzie sinned so deeply as to merit professional extinction? For that is really the punishment which it is attempted to mete out to him. We do not approve of what Sir Morell Mackenzie has done. He has undoubtedly offended against some of the best traditions of medicine. But we do say that the punishment is in excess of the offence. We do not say this for Sir Morell Mackenzie's sake, but for the sake of the honour of the medical profession. A blunder has been committed by the Edinburgh professors. An error of judgment has been treated as a crime. The result is that the sympathy of the world, which should have been on the side of the judges, that is of the medical profession, is transferred to the offender. Sir Morell Mackenzie has done nothing to justify the whole medical profession in rising up against him. The demonstration is a thousand times greater than the occasion. An army of a hundred thousand men has been called out to quell a village riot. The leaders of the medical world have made the profession ridiculous in the eyes of the public. Lawyers are never seen in such undignified attitudes; members of Parliament would scorn to place themselves in such false and foolish positions. Over and over again the leaders of the medical profession have amused and amazed the world by the spectacle of hundreds of learned men moved to expressions of the profoundest horror by some question of mere "professional form;" whilst really serious moral lapses on the part of more or less conspicuous men have been passed over in silence. The fact is that, whether they wish to do so or not, medical men constantly give to the world the impression that their work, their words, and their religion all centre in the one word "etiquette." By "etiquette," and by nothing else, is a doctor saved or damned. We protest against the action of the leaders of the profession in Edinburgh on behalf of those members of the profession who are young enough and have soul enough to be men as well as doctors.

